# Supplementary material for: Preferential apical infection of Caco-2 intestinal cell monolayers by SARS-CoV-2 is associated with damage to cellular barrier integrity: Implications for the pathophysiology of COVID-19
Source: PLoS One. 2025 Feb 10;20(2):e0313068. doi: 10.1371/journal.pone.0313068 (PMC11809792; doi:10.1371/journal.pone.0313068)

Supporting information

S1 Fig. Microscopy analysis of the PAS (Periodic Acid-Schiff) coloration for the mucin detection in the Caco-2/HT29 coculture (Scale bare: 10 µm, n=2).

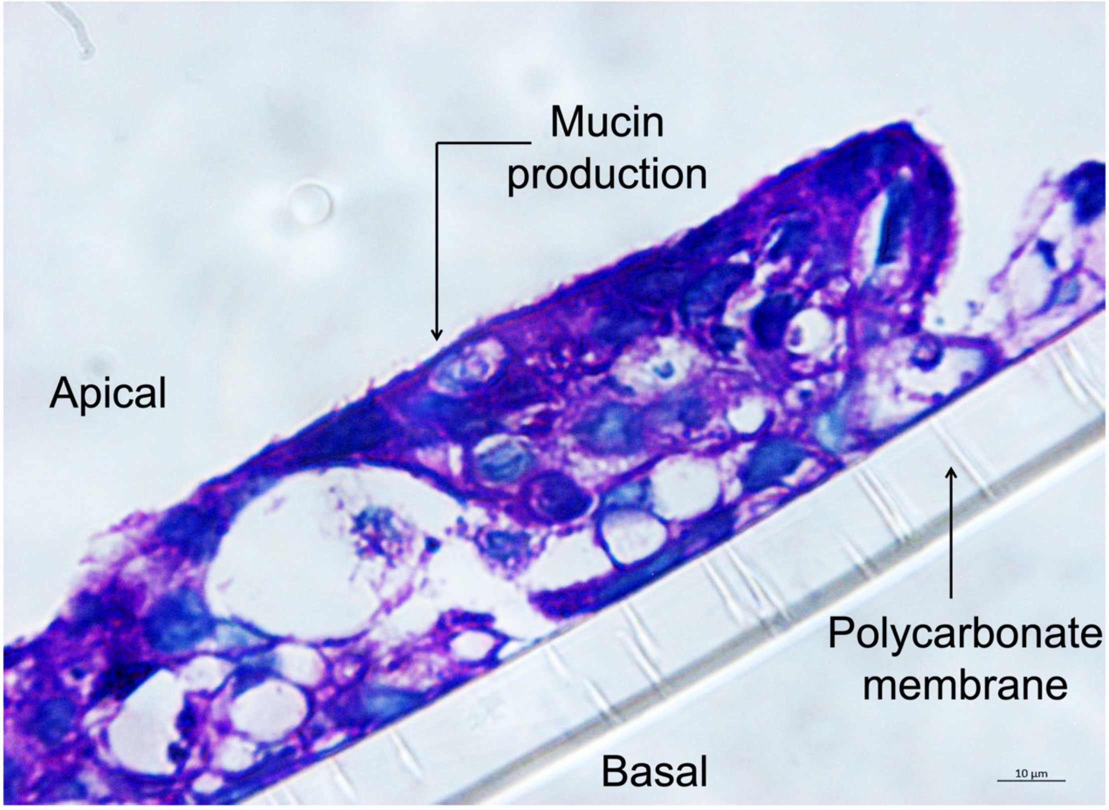

Supplement: S1 Fig — (PDF) [file pone.0313068.s001.pdf]
